# Secondary structure of the segment 5 genomic RNA of influenza A virus and its application for designing antisense oligonucleotides

**DOI:** 10.1038/s41598-019-40443-7

**Published:** 2019-03-07

**Authors:** Paula Michalak, Marta Soszynska-Jozwiak, Ewa Biala, Walter N. Moss, Julita Kesy, Barbara Szutkowska, Elzbieta Lenartowicz, Ryszard Kierzek, Elzbieta Kierzek

**Affiliations:** 1 Institute of Bioorganic Chemistry Polish Academy of Sciences, 61-704 Poznan, Noskowskiego 12/14, Poland; 2 Roy J. Carver Department of Biophysics, Biochemistry and Molecular Biology, Iowa State University, Ames, IA 50011 USA

## Abstract

Influenza virus causes seasonal epidemics and dangerous pandemic outbreaks. It is a single stranded (−)RNA virus with a segmented genome. Eight segments of genomic viral RNA (vRNA) form the virion, which are then transcribed and replicated in host cells. The secondary structure of vRNA is an important regulator of virus biology and can be a target for finding new therapeutics. In this paper, the secondary structure of segment 5 vRNA is determined based on chemical mapping data, free energy minimization and structure-sequence conservation analysis for type A influenza. The revealed secondary structure has circular folding with a previously reported panhandle motif and distinct novel domains. Conservations of base pairs is 87% on average with many structural motifs that are highly conserved. Isoenergetic microarray mapping was used to additionally validate secondary structure and to discover regions that easy bind short oligonucleotides. Antisense oligonucleotides, which were designed based on modeled secondary structure and microarray mapping, inhibit influenza A virus proliferation in MDCK cells. The most potent oligonucleotides lowered virus titer by ~90%. These results define universal for type A structured regions that could be important for virus function, as well as new targets for antisense therapeutics.

## Introduction

Influenza virus causes global epidemics each year and infrequent, but dangerous pandemic outbreaks. According to the WHO, seasonal flu is responsible for up to 650000 deaths yearly^1^. Currently, there are only a few treatments or protections available against influenza. Vaccines, containing inactivated or attenuated virus or pure viral proteins must be reformulated and administered annually; efficacy levels can vary greatly depending on what strains actually circulate^1–3^. The currently available drugs target viral surface proteins: these include M2 ion channel blockers and neuraminidase inhibitors^4^. However, resistance to small-molecule therapeutics is growing^4^. There is a great need to find other approaches for combating influenza virus. One promising new approach focuses on influenza RNA as a therapeutic target.

Influenza is an RNA virus with a single stranded, negative-sense viral genome (v)RNA. vRNA is divided to eight segments^5^; which, in virions, exist in viral ribonucleoprotein (vRNP) complexes that contains (beside vRNAs) multiple NP and polymerase proteins. Each vRNP complex is an independent replication-transcription unit that uses its heterotrimeric viral RNA-dependent RNA polymerase. vRNA is a template for mRNA and complementary (c)RNA that is used as a template for replicating vRNA^5–7^. RNA is used throughout the viral replication cycle, highlighting its central role in influenza biology. Cryo-electron microscopy has provided important insights into the global 3D vRNP structure^8,9^. Later, using high-throughput sequencing of RNA isolated by crosslinking immunoprecipitation (HITS-CLIP), the vRNA binding profiles of NP was identified for two strains^10^. These studies revealed that vRNA can loop out from complex with NP and potentially form secondary structure, even in RNP. In cells, viral RNA structure could have additional roles depending on the step of the replication cycle.

RNA function is closely related with its structure. For influenza virus structure-function relationships are emerging as new studies continue to discover roles for RNA structure. Initial bioinformatics scans revealed the possibility of finding conserved structural motifs across both (−) and (+) strands^11–13^. Multiple structured regions have been confirmed experimentally^14–17^ and several were confirmed as functional by additional experiments^16,18^. So far, only the secondary structures of segments 7 and 8 vRNAs were experimentally determined *in vitro* (the full genome sequence)^19,20^. Knowledge of vRNA secondary structure is crucial for understanding virulence and developing inhibitors; thus, in this study, the secondary structure of the segment 5 vRNA (vRNA5) was modeled. This segment encodes NP protein, which plays several roles during the viral life cycle, including vRNP formation, transport of vRNA-polymerase complex to the nucleus, viral replication, and virion packaging^5^. Because NP protein is abundant—compared to other viral proteins—and appears in almost each step of virus propagation, it draws the attention of scientists both in terms of understanding its functions and as a potential target of new therapies. Recently, several RNA structural motifs were proposed for the vRNA5^21^. Other studies of vRNA5 indicated that select regions interact more weakly with NP, therefore allowing them to fold into RNA motifs^22^. The functions of vRNA5 motifs were verified by mutagenesis: e.g. synonymous mutations designed to alter the predicted RNA structures in these low-NP-binding regions impact genome packaging and result in virus attenuation, whereas control mutations or mutagenesis of NP-bound regions have no effect^22^. NP binding profiles also confirm previous results and showed that there are differences NP associations between strains^10,23,24^. This interesting finding could explain different patterns of interactions between vRNPs through RNA found by others. Recent data reveals highly structured genomes with space to allow a redundant network of vRNA-vRNA interactions necessary for packaging^25^.

Herein, the secondary structure of entire segment 5 vRNA (vRNA5) is determined for the first time. The basis of prediction was chemical mapping supported by isoenergetic microarray mapping, free energy minimization and bioinformatics analysis. The data presented complements previous results regarding the suggestion of existence of vRNA5 structural motifs by presenting an experimentally-informed global view of whole segment structure, which shows conservation across a variety of strains. Furthermore, results showing oligonucleotide accessibility can act as leads for designing antisense oligonucleotides: indeed: several oligonucleotides are shown to be active vs. vRNA5 and inhibit virus proliferation.

## Results

### Chemical mapping of segment 5 vRNA

**Figure 1:**
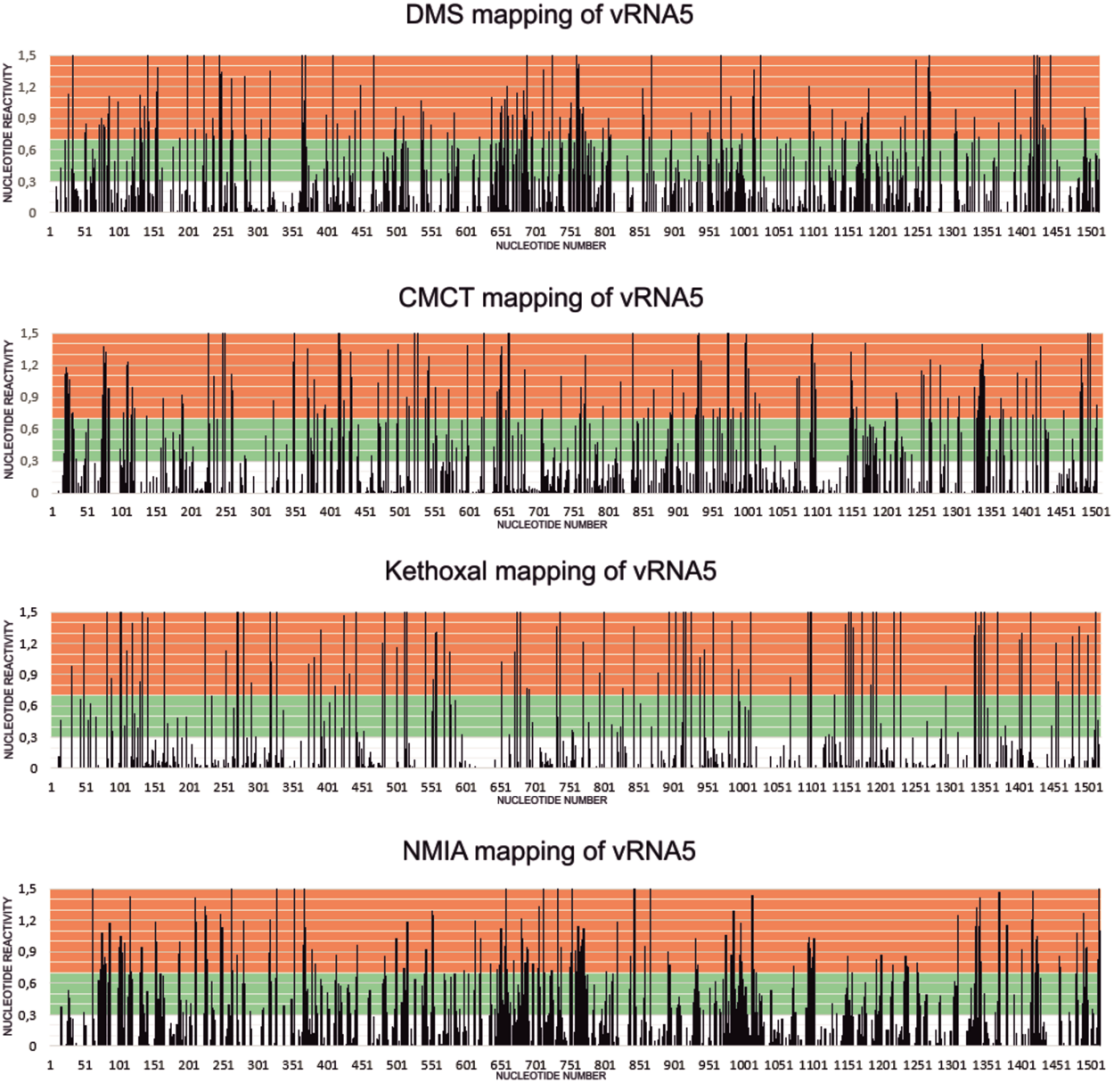
The influenza A virus vRNA5 nucleotides reactivity diagrams. The vRNA5 chemical mapping experiments were performed at 37 °C with DMS, CMCT, kethoxal and NMIA. On graphs low reactivities (values <0.300) were marked with white, medium reactivities with green (values 0.300–0.700) and high reactivities (values ≥0.700) with orange shadow.

Chemical mapping enables one to obtain information about single stranded regions of RNA that are accessible to modifying reagents and double stranded regions that are not. Influenza virus vRNA5 secondary structures were studied using chemical mapping with NMIA, DMS, CMCT and kethoxal. NMIA modifies all accessible nucleotides at the 2′OH whereas DMS methylates A and C (N1 and N3 respectively), CMCT methylates N3 of U and kethoxal methylates G at N1 and N2^26–28^. Before mapping, appropriate folding conditions were optimized to yield a single RNA conformation, as judged by non-denaturing agarose gel (Fig. S1). Results of structure mapping at 37 °C (Fig. 1) show that 134 nucleotides were strongly modified with DMS (17.7% of adenosines and cytidines of vRNA5). There were 282 strong and medium uridine modifications with CMCT (56.9% of all U). Kethoxal modified strongly 101 and moderately 51 guanosines (48.1% of all G). Whereas, 169 and 274 nucleotides were strongly and moderately modified by NMIA, respectively. Susceptibility of vRNA5 for reaction with NMIA was rather evenly distributed, but there are regions more exposed, with continuous reactivity. The most reactive regions in vRNA5 were 18–30 nt, 71–87 nt, 635–688 nt, 748–771 nt, 1326–1341 nt, 1409–1425 nt. Less reactive regions were 1102–1200 nt and 590–610 nt. Premature termination of reverse transcription observed in 1074 nt to 1115 nt and also 1140 nt and 860 nt showed evidence of highly structured regions (Figs 1, S2).

### Influenza A virus segment 5 vRNA secondary structure

**Figure 2:**
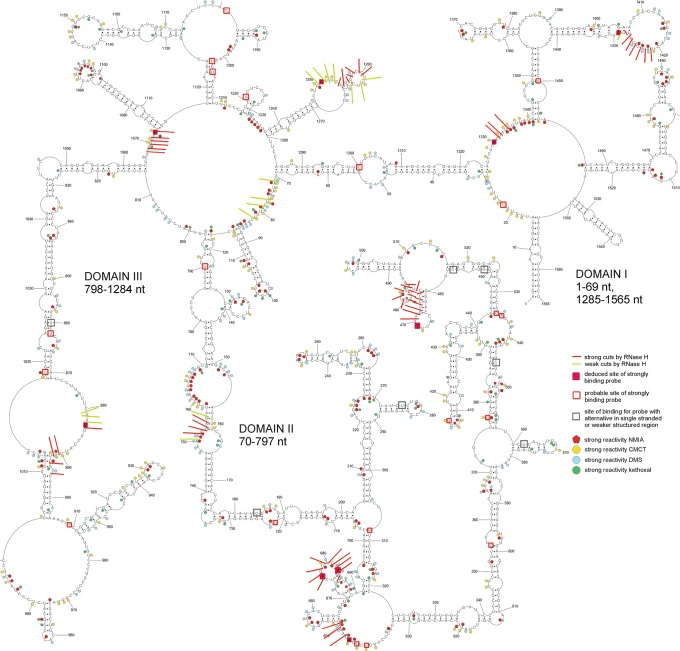
Secondary structure of influenza A vRNA5 predicted by RNAstructure 5.7 using experimental data from 37 °C as constraints. Strong DMS, CMCT and kethoxal modifications, as well as SHAPE reactivities converted to pseudo-free energies were used. Beside strong also medium reactivity to DMS, CMCT, kethoxal, results from RNase H cleavage in presence of DNA oligonucleotides (neither used in modeling) and microarrays mapping results are annotated. The numbering of vRNA5 is from its 5′ end.

Chemical mapping results from 37 °C were used as constraints in vRNA5 secondary structure prediction with RNAstructure 5.7 (*Materials and Methods*). The vRNA5 structural model with the lowest Gibbs free energy was selected as the final model structure (Fig. 2). vRNA5 is highly structured and three domains can be distinguished. Domain I contains motifs between 1–69 nt and 1285–1565 nt. Domain II consists of a region spanning 70–797 nt and domain III 798–1284 nt. Domain I contains (1–16 nt/1565–1551 nt), which forms the panhandle motif that is conserved across orthomyxoviruses^11,29^. The most reactive regions (70–85 nt, 635–690 nt and 758–771 nt) are in domain II. There are 23 hairpins present in the vRNA5 secondary structure overall. Some of them are strongly mapped with modifying reagents in loop regions: e.g. nts 87–115, 493–502, 409–418, 1074–1115, 1136–1160, 1404–1429 and 1483–1497. The longest helix motif (811–871/1017–1064 nt) was identified in domain III and it is compatible with the few modifications observed in this region (Fig. 2, Supplementary Data 1).

vRNA5 structure was also mapped at 23 °C to evaluate if lower temperature would change folding. Reactivities of nucleotides in lower temperature were mostly the same (Supplementary Data 1). Predicted vRNA5 secondary structure using chemical mapping data from 23 °C reveal changes in several regions which, in general, could be grouped to 2 clusters: first in Domain I and second in Domain II/III (Fig. S3). The main difference in vRNA5 secondary structures is the absence of the panhandle motif at 23 °C. All motifs that vary with temperature are marked in pink on the 23 °C vRNA5 secondary structure (Fig. S3).

### Isoenergetic microarray mapping estimates of segment 5 vRNA accessible sites for oligonucleotides

Isoenergetic microarray mapping is a method that uses RNA binding to short oligonucleotides to probe RNA secondary structure. Isoenergetic microarrays used in this study were comprised of penta- and hexa-nucleotide probes that bind to target RNA isoenergetically: i.e. binding strength is independent of the sequence. To generate isoenergetic probes, 2′-O-methyl, LNA and 2,6-diaminopurine modifications were incorporated in specific positions in the oligonucleotide^30,31^. Universal microarrays (applicable to any RNA target) with 877 different penta- and hexanucleotides probes were used (Supplementary Data 2).

**Figure 3:**
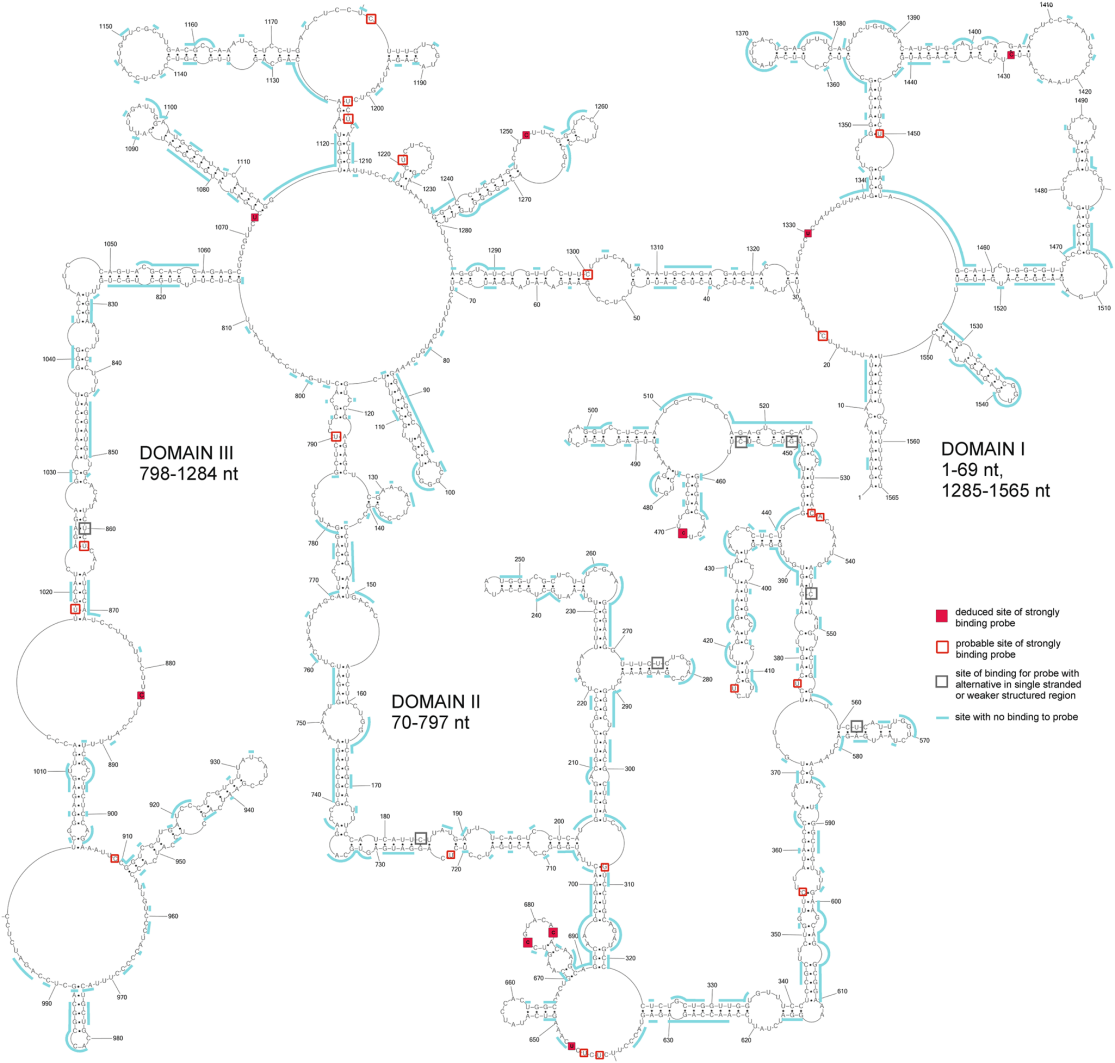
Microarrays mapping results for vRNA5. Sites that strongly bind complementary probes and sites that are not accessible for probes are marked on vRNA5 secondary structure.

Prior to microarray mapping, vRNA5 was radioactively labeled during *in vitro* transcription and, after folding, hybridization to isoenergetic microarrays was performed. Hybridization to isoenergetic microarrays gave whole vRNA5 screening with complementary, mostly step-by step, short probe. Single-stranded and labile regions in studied RNA are accessible to probes, whereas double-stranded thermodynamically stable structures are not^32^. These results complement and facilitate RNA secondary structure mapping methods and structure predictions. Additionally, microarray mapping results directly show regions that bind short oligonucleotides or those that do not bind. Probe binding sites were identified in all three domains of vRNA5 (Figs 2, S4 and Table S4). Some probes can bind to more than one site in vRNA5, which are designated as alternative binding sites. Alternative binding sites are predicted using RNAstructure (*bimolecular binding* mode, Table S4). An additional approach was used for final determination of probe binding sites. Binding sites denoted as “deduced” are confirmed with RNAse H assay results. “Probable” binding sites are sites that are energetically and structurally favorable. Binding sites, including probable ones are: 22, 308, 355, 377, 415, 534, 535, 642, 644, 646, 677, 683, 722, 790, 862, 883, 909, 1018, 1073, 1181, 1203, 1205, 1219, 1251, 1300, 1330, 1429, 1450. Probes with more than one binding sites have at least one in structurally reasonable, as single stranded and/or flexible, vRNA5 region, which confirmed the proposed secondary structure^31^. No binding was observed in 1074–1179 nt (domain III), which is the largest region in vRNA5 not accessible to probes. All RNA fragments that do not bind to isoenergetic probes are marked on Fig. 3. It is notable that non-binding probe regions that constituted at least 5 sites are in long helixes of proposed secondary structure and in two cases include three-nucleotides hairpin loop (Fig. 3). These results help to define structured regions in vRNA5 and complement data which define unstructured, accessible sites.

### RNAse H assays confirm selected oligonucleotide binding sites in segment 5 vRNA

An RNAse H assay was used as additional approach for the estimation of vRNA5 accessibility to oligonucleotides in selected regions and to clarify interpretation of microarray mapping results for these regions. In this study, 14 DNA oligonucleotides were designed (Table S5) to evaluate and confirm microarray probe binding.

All RNase H cleavage sites (strong and weak) in the vRNA5 secondary structure are presented on Fig. 2 and in Table S5. The results confirm both accessible and inaccessible vRNA5 regions. No cleavage was observed for H9 oligonucleotide, which was complementary to 1102–1109 nt. This is reasonable because this region was in the helix of a hairpin that spans 1074–1115 nt. Cleavage sites were mainly in single-stranded regions (except 470–484 nt, 753–755 nt and 1255–1257 nt) in expected regions. Cleavage sites are in agreement with regions that are heavily chemically mapped. The results of the RNase H assay corroborate isoenergetic microarray biding sites at 469, 646, 677, 683, 883, 1073, 1251, 1429 and 1330 nt (Table 1). In the RNase H assay results, some hydrolysis sites were identified at helices: in hairpin 460–476 nt and 1255–1266 nt and short helixes near bulges (753–757/156–160, 892–894/1011–1013). This indicates that those structures can be easy opened with oligonucleotides (e.g. via strand invasion).

**Table 1:** Deduced strong binding sites in vRNA5 of isoenergetic microarray probes.

Binding site^a,b^	Deduced binding sites^c^	Probe sequence^d^	Strength of probe binding^e^	Predicted ΔG°_37_ of probe/ vRNA5 duplex (kcal/mol) for confirmed binding sites^f^	RNase H cleavage sites
22/184/355/880/883/1251/1280/1300/1429	883 1251 1429	dDgDdg	S	−9.24 (883, 1251, 1429)	880–882 (w) 883 (s) 1250–1253 (w) 1254–1260 (s) 1261 (w) 1421–1427 (s) 1428 (w)
256/457/469/546/814/1181	469	dDgDgg	S	−10.03 (469)	470–484 (s,w)
677	677	DcGgDg	S	−10.81 (677)	676–684 (s)
275/642/644/790/860/1073/1203/1221/1328/1330	1073 1330	dGdGdg	S	−10.71 (1073) −12.84 (1330)	1067–1071 (s) 1073 (s) 1331–1332 (s) 1334 (s)
534/683	683	gUgUgg	S	−9.77 (683)	676–684 (s)
377/415/562/646/722/862/1205/1450	646	uGdGdg	S	−12.22 (646)	647 (s) 649–650 (s)

^a^Possible complementary binding sites of probes, ^b^sites are denoted by the middle nucleotide of the complementary RNA region; ^c^deduced binding site for probe by comparison with DNA induced RNase H cleavage results; ^d^nucleotides in capital letter (G, U, D) are 2′-O-methyl-RNA nucleotides, lower case letters (c, g, u, d) are LNA nucleotides, D and d are 2,6 – diaminopurine (2′-O-methyl type or LNA, respectively); ^e^binding was considered strong (S) when the integrated intensities were ≥1/3 of the strongest intensity. Hybridization condition: buffer 1, 37 °C; ^f^ΔG°_37_ calculated as modified probe/RNA duplex^67,68^, in parenthesis, the site of binding for which calculation was done; ^g^(s) – strong RNase H cleavage, (w) – weak RNase H cleavage.

### Base pair probabilities of the vRNA5 secondary structure model

**Figure 4:**
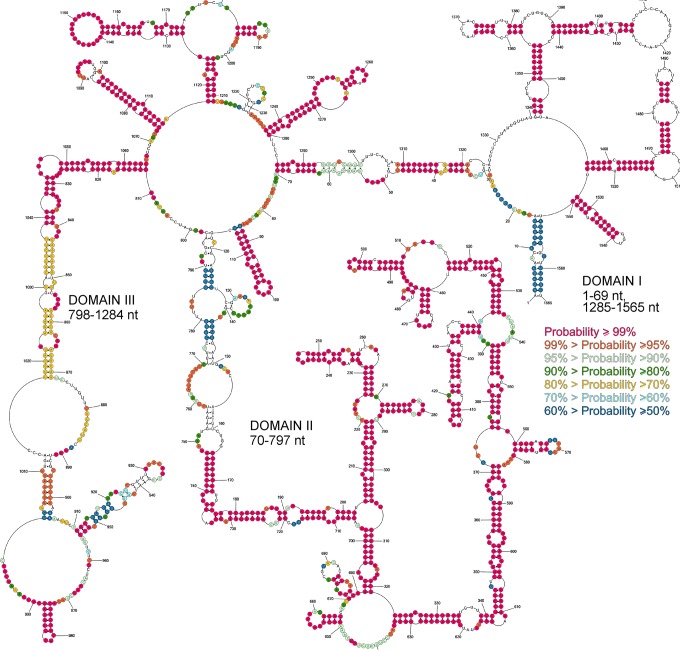
The probability of forming base pairs and single-stranded regions in vRNA5. The probability was calculated with the RNAstructure web server (The Predict a Secondary Structure server). Probability lower than 50% is not marked on the structural model. The partition function calculation incorporated restraints from strong reactivity of DMS, CMCT, kethoxal and SHAPE (converted to pseudo-energy).

The base pair probabilities of the vRNA5 secondary structure model were estimated as described in the Materials and Methods; single stranded nucleotide probabilities were also estimated. In the calculation, experimental structure mapping data were incorporated as constrains. The calculated probability data give information about structure prediction quality and identify well-defined structural motifs. Results are presented on (Fig. 4). The most probable (≥99%) motifs are in domain II (165–748 nt). Most vRNA5 hairpins are very probable, except the hairpin 128–140 nt and motif 910–954 nt. Also, the region 843–1038 nt is less probable. In these regions other, less thermodynamically favorable, structural motifs can be formed (Fig. 4).

### Conservation of segment 5 vRNA secondary structure model for type A

**Figure 5:**
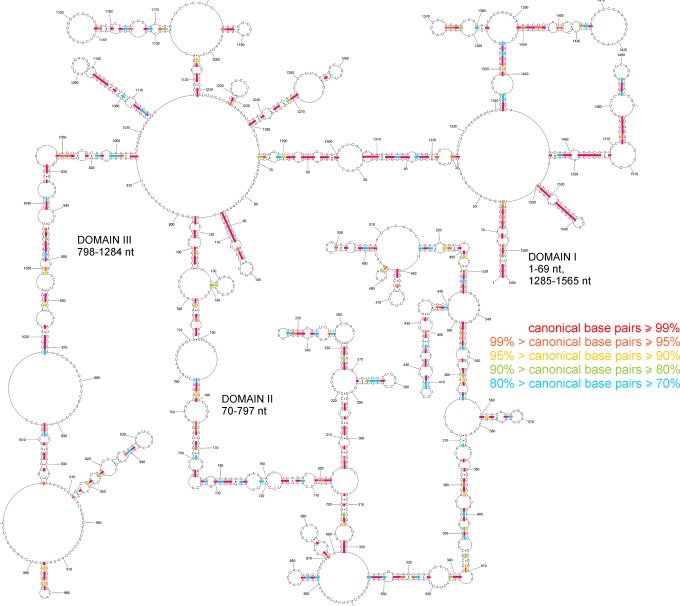
Conservation of vRNA5 secondary structure in type A of influenza viruses. Colors indicate percentage of canonical base pairing preserved across influenza A sequences for vRNA5 and was performed on 18500 sequences.

Structure conservation of the proposed secondary structure model was analyzed using all available influenza A sequences (18500 sequences, *Material and Methods;* Supplementary Data 3). The proposed secondary structure of vRNA5 is 87% conserved throughout influenza A sequences (Fig. 5, Supplementary Data 3); suggesting that it may play roles throughout influenza A strains. The secondary structure model is also supported by potential compensatory (double) and consistent (single) mutations that preserve base pairing across type A influenza strains (Supplementary Data 3).

Consistent with its known functional importance in replication^29,33^, the panhandle motif (1–16/1551–1565 nt) was highly conserved (97.5%). In addition to the panhandle structure, ca.130 nt from 5′ and ca. 100 nt from 3′ end of the vRNA5 sequence also encodes packaging signals^34^. Interestingly, conserved hairpins were found in both regions: spanning nts 87–115 and 1483–1497. Both hairpins are conserved (99.5 and 87.2% conserved, respectively). Variations in sequence could preserve base pairing (particularly in nts 87–115), however, numerous inconsistent mutations were observed in the less well conserved (latter) hairpin (Fig. 5, Supplementary Data 3).

Besides the packing signal regions there are other conserved motifs across vRNA5. The conservation of hairpin 974–988 nt is 96.9%. Other conserved motifs are located within the highly structured region of 1065–1281 nt. The conservation of this region is 83.5% on average; however, the conservation of base pairing in this region is heterogeneous – it is below average for the stem region of hairpin 1080–1083 and is very high (up to 100%) for base pairs 1084/1114 nt-1088/1099 nt. The vRNA5 base pair conservation of 86.8% is calculated for helix 1117–1125/1211–1203 nt, whereas the structural motif in region 1127–1173 nt is conserved 84.4%. The conservation of 460–476 nt hairpin is 83% and for hairpin 477–484 nt is 95.6% (Fig. 5).

### Inhibition of influenza virus proliferation by antisense oligonucleotides targeting vRNA5

**Figure 6:**
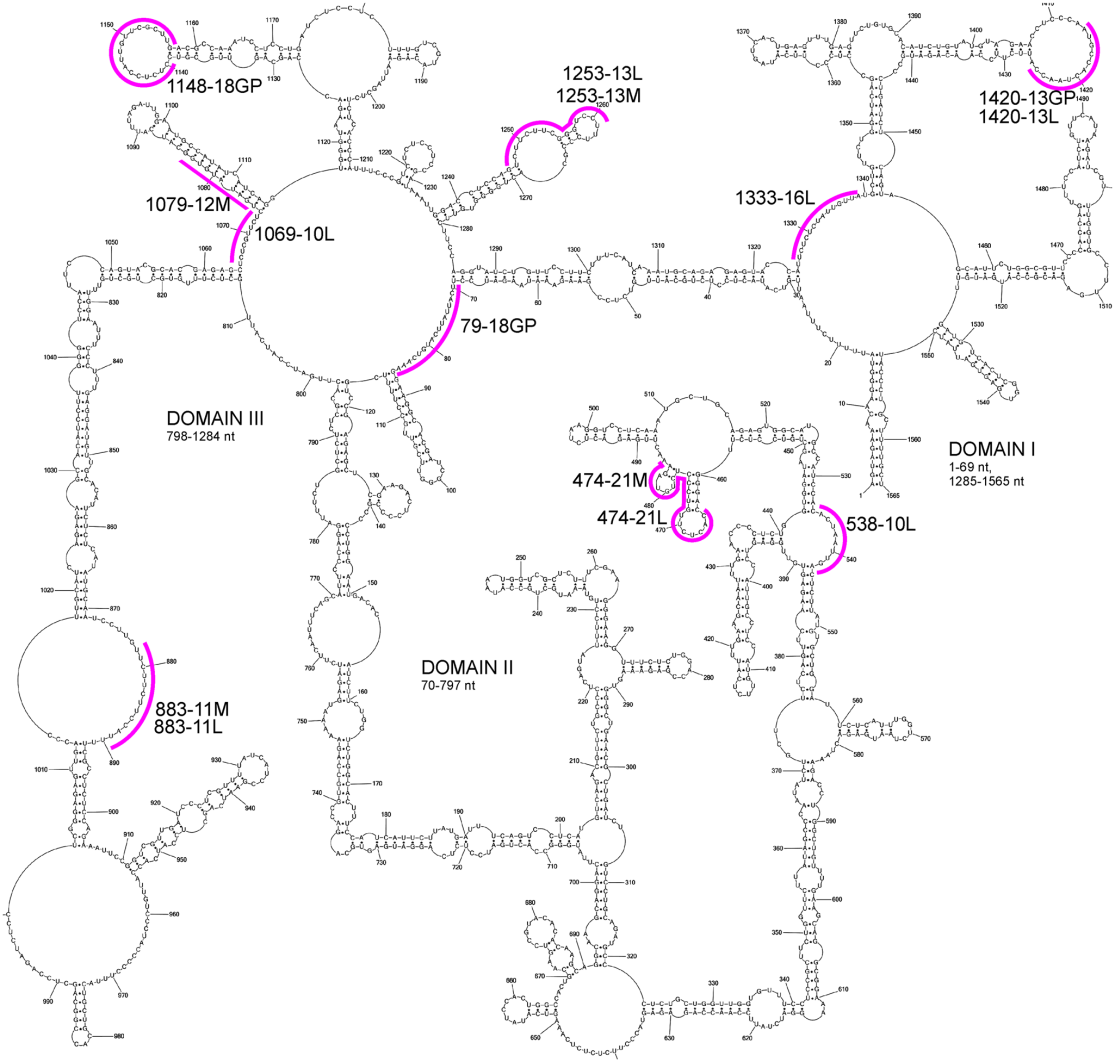
Binding sites of antisense oligonucleotides marked on vRNA5 secondary structure. Complementary regions for each ASO is marked by pink line. Name of oligonucleotide comes from designed binding site (the middle nucleotide of the five nucleotides complementary in the RNA - first part of name) and length (second part of name). M or L in ASO name means 2′OMeRNA or 2′OMeRNA-LNA, respectively.

**Figure 7:**
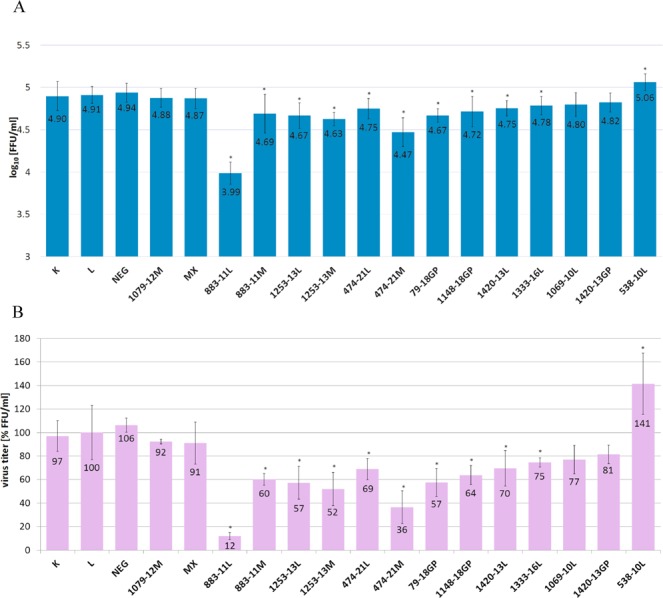
Antiviral activity of ASOs in MDCK cells against A/California/04/2009. Antiviral activity was analyzed by IFA and calculated virus titer is presented in two scales: (**A**) log_10_ [FFU/ml] and (**B**) [% FFU/ml]. The final ASO concentration was 0.5 μM. The mean was calculated from three independent experiments, each containing three technical repeats (9 data points) and the standard deviation is shown. K is a virus titer in untreated MDCK cell line infected with IAV, L - virus titer from cells treated with Lipofectamine 2000 only (LPF). The remaining labels indicate virus titer from cells treated with particular ASO. NEG and MX are negative control oligonucleotides. Statistics were calculated using a two-tailed T-test (p < 0.01). Statistically important results are marked with *.

Based on the modeled secondary structure, microarray mapping and RNase H assay results, 16 antisense oligonucleotides (ASOs) targeting vRNA5 were designed to inhibit influenza virus replication. Additional negative control oligonucleotides were used: NEG (ASO non-complementary to vRNA5)^35,36^ and MX (mixmer of nucleotides of 474–21M) (Table S6, Fig. 6) and also 1079–12M, which is complementary to a double stranded region of the vRNA5 structure and is not expected to inhibit virus proliferation. All oligonucleotides were 2′OMeRNA and several have additional LNA modifications. MDCK cells were transfected with antisense oligonucleotides (at 0.5 μM concentration), then infected with A/California/04/2009 (H1N1) strain and for calculation of virus titer IFA (Indirect Immunofluorescence Assay) was first performed. Five antisense oligonucleotides 883–11L, 474–21M, 1253–13M, 1253–13L and 79–18GP inhibited IAV replication by more than 40% (Fig. 7). The most effective oligonucleotide was 883–11L, which inhibited influenza virus replication by 88% (Fig. 7). Oligonucleotide 883–11L was 5-fold more effective than the same oligonucleotide sequence without LNA modifications 883–11M. Whereas, oligonucleotide 474–21M resulted in 64% inhibition, which was 2-fold more inhibiting than oligonucleotide 474–21L containing LNA modification (31%).

**Figure 8:**
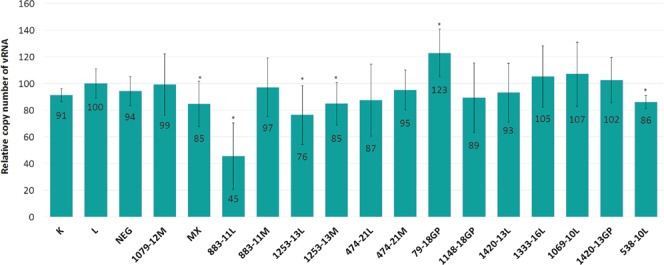
Antiviral activity of ASOs in MDCK cells against A/California/04/2009. Antiviral activity was analyzed by qRT-PCR. The final ASO concentration was 0.5 μM. The mean was calculated from three independent experiments each containing three technical repeats, additionally each repeat was analyzed by three independent qRT-PCR reactions (27 data points) and the standard deviation is shown. K is a virus titer in untreated MDCK cell line infected with IAV, L - virus titer from cells treated with Lipofectamine 2000 only (LPF). The remaining labels indicate virus titer from cells treated with particular ASO. NEG and MX are negative control oligonucleotides. Statistics were calculated using a two-tailed T-test (p < 0.01). Statistically important results are marked with *.

For selected ASOs, qRT-PCR was used to determine the levels of vRNA reduction. The results were compared with the standard curve and are presented in Fig. 8. The lowest vRNA level (45%) was observed for 883–11L. This result is in line with IFA results, where 883–11L was the most inhibiting oligonucleotide (Fig. 7). The statistically lowering of vRNA level was also observed with oligonucleotides 1253–13L (76%), 1253–13M (85%), and 538–10L (86%).

The most inhibitory ASOs were tested for cytotoxicity at concentrations of 0.5 μM using MTT assay. The ability of NAD(P)H-dependent cellular oxidoreductase enzymes to function is measured, this reflects cell viability (Fig. S5). In general, antisense oligonucleotides are not cytotoxic.

## Discussion

In the presented study, the secondary structure of entire vRNA5 of IAV was determined based on *in vitro* chemical mapping coupled with free energy minimization and bioinformatics sequence/structure analysis. vRNA5 is highly structured with distinguishing motifs and domains. Evidence of single stranded and accessible regions were showed by reactivity of nucleotides to four mapping reagents, binding to isoenergetic probes and targeted RNase H cleavage in the presence of selected DNA oligonucleotides (Fig. 2). In general, most of the predicted loops in the vRNA5 secondary structure are heavily chemically mapped. Whereas single-stranded regions of 1382–1392 nt, 780–786 nt, 871–888 nt, 1174–1182 nt and 954–966 nt have no or few modifications only. The region of 780–786 nt is, however, accessible for RNase H hydrolysis and strong isoenergetic microarray probe binding was observed. For 1174–1182 nt region, a probable site for a strongly binding probe was also detected. Other single stranded regions may be inaccessible due to possible tertiary interactions (Fig. 2).

Chemical and isoenergetic microarray mapping also indicated regions that are involved in base pairing (Supplementary Data 1, Fig. 3). Predicted helixes in vRNA5 secondary structure are confirmed by no binding to microarray probes, therefore are in agreement with chemical mapping data (Figs 2 and 3). Several hairpin loops and bulges are only partially accessible (e.g. 1366–1372 nt., 1089–1098 nt., 1140–1156 nt. and 634–550 nt) suggesting interactions within the loops or with another RNA fragments. Premature termination of reverse transcription was observed in hairpin 1074–1115 nt region (Fig. 2). This is likely due to the high thermodynamic stability of this structure. Termination of transcription was also detected at 1140 nt; additionally, no reverse transcription product was obtained from the primer that was complementary to 1092–1116 nt in vRNA5 (primer P8 and poor efficiency with primer P7, Table S2). These findings are additional evidence for the existence of this stable hairpin. Flanking secondary structure motifs and tertiary interactions in this domain likely contribute to its thermodynamic stability and poor accessibility to oligonucleotides.

Structural conservation of vRNA5 model is on average high for type A strains (87% of base pairs are conserved) (Fig. 5). Conservation of base pairs across A strains despite of changing sequence (compensatory and consistent mutations) additionally confirm secondary structure model of vRNA5 (Supplementary Data 3). The structural conservation for domains I, II and III is 92%, 86%, 85% respectively and several motifs are even more conserved. Such motifs are interesting because structure universality and preservation across strains indicates biological function.

In the proposed secondary structure model there are motifs that were previously predicted by Gultyaev *et al*. to be possible both in the (−) and (+) strand as a “mirror structures”^21^. Among those motifs, there is a hairpin at 1527–1550 nt (16–39nt in + RNA) which is located within the packaging signal, and it is possible that this motif may be important for packaging. The presence of the 1527–1550 nt hairpin in the vRNA5 structure is very conserved across strains of type A (Fig. 5). Also, hairpin 974–988 nt (576–592 nt in +RNA numbering) with high base pairs probability for type A was identified previously.

For the region 1462–1476 nt (89–105 nt in +RNA numbering) Gultyaev *et al*. predicted the presence of a hairpin^21^. Although the sequence of A/Vietnam/1203/2004 (H5N1) can form this structure, our model of the secondary structure of this region is different, also the chemical mapping profile excludes the formation of this hairpin (Fig. 2). Similarly, the hairpin in region 626–643 (region 922–938 nt in (+)RNA) is excluded by chemical mapping. Although the presence a hairpin 36–90 nt (1476–1530 nt in +RNA numbering) has been proposed previously; this motif does not appear in our vRNA5 secondary structure model. Interestingly, previous experiments that aimed to disrupt the secondary structure of this motif had no effect on replication^21^. These mutations are located within single-stranded nts in our model: a bulge at nt 39 or a loop (for nts 85 and 87) (Fig. 2), so they cannot affect secondary structure, which is consistent with no replication effect in this previous study^21^.

vRNA5 nts 90–130 were previously predicted to form a pseudoknot^21^. Secondary structure studies revealed that the potential pseudoknot base pairs (124–130/96–102 nt) had strong chemical mapping signals that preclude their formation *in vitro* (Fig. 2). It is possible that the presence of a pseudoknot in the vRNA5 structure is strain-specific. In this region the hairpin 87–115 nt is highly probable in the base pair probability calculation, but structures predicted within fragments 117–150/771–797 nt are less probable and the presence of other alternative structures cannot be excluded (Fig. 4).

A secondary structure model of (+)RNA5 based on A/Vietnam/1203/2004 (H5N1) strain was determined previously^36^. It is interesting to compare secondary structure of vRNA5 (Fig. 2) to (+)RNA5^36^. The reverse complement of the sequence yields the possibility of forming “mirror structures” in both strands; conversely, any structural differences could be important for RNA strand recognition by virus and host factors. Indeed, vRNA5 and (+)RNA5 have several similar structural motifs: e.g. 1341–1454 nt (107–227 nt in (+)RNA), 89–113 nt (1453–1477 nt in (+)RNA), 460–476 nt (1090–1106 nt in (+)RNA, 1243–1273 nt (293–323 nt in (+)RNA), 1472–1506 nt (60–94 nt in (+)RNA), 1531–1545 nt (21–35 nt in (+)RNA) (Fig. S6)^36^. Most structural motifs located in packaging region are “mirrors” of complementary regions structures in the (+)RNA; however, only vRNA segments are packaged into progeny virions, while cRNAs are degraded in the host cell. In the process of packaging, M1 binds only vRNP. cRNP is not recognized by M1 and, as a result, the transport of vRNP and cRNP in the cytosol differs^37^. Specific binding of M1 is responsible for packaging of vRNP into virions and unique features should be used. Structure similarity in 5′and 3′ ends regions could be needed for NP and polymerase binding to form vRNP and cRNP complexes. This suggest that not only sequence/secondary structure in the ~150 nt fragments at 5′ and 3′are important to packaging, but internal regions may also have an impact on the process. This is consistent with observations for other vRNA segments, where internal regions are responsible for vRNA/vRNA intermolecular interactions^38–40^.

Knowledge about naked vRNA structure is needed to identify thermodynamically stable structure motifs that could preferentially form and have biological function. *In vitro* experiments in native buffer with the absence of proteins allows one to study exclusively RNA properties and enables clear interpretation of results. Rigid and flexible regions of RNA structure, and the possibility of rearrangements could be important for different RNA functions. Also, they can be targets for future drugs which disturb or block RNA structural motifs.

Lowering temperature to 23 °C causes rearrangements in mainly two parts of the structure, but overall the differences are small (Fig. S3). This lower temperature was used because there are influenza strains that can effectively replicate in low temperature (e.g. cold adapted viruses) as well as others in high temperature (e.g. avian strains)^41,42^. In general, influenza can infect different host species and replicate over a large range of temperatures^41,42^. The temperature-stimulated differences observed in the panhandle are consistent with hypothesized functions connected to alternative folds of the 5′ end 3′ ends of the vRNA (such as the *hook and cork screw* conformations)^33,43,44^. Likewise, other dynamic regions could also be important in the virus replication cycle. For example, the rearrangements in domain II, which includes a packaging signal sequence may be functional.

In the cell vRNA5 is coated with nucleoprotein (NP). It was shown, however, that NP monomers bind irregularly, which allows vRNA structural motifs to be formed^10,22^. This allows for a vRNP structural model where vRNA loops out from the protein complex and folds to create functional structural motifs—possibly in a dynamic manner^10^. From the studies of two vRNA5 strains (A/WSN/1933 (H1N1) and A/California/07/2009 (H1N1)), similarities were noticed in NP binding profiles. There are also differences that are specific to each strain, especially in proportion of bound NP to different vRNA regions^10^. In general, in regions located within the promoter and packaging signals at the 3′ and 5′ ends, NP binding is reduced and the major NP binding sites can be identified within the internal region. Significant lowering of NP binding for both A/WSN/1933 (H1N1) and A/California/07/2009 (H1N1) were noticed in internal regions of vRNAs, such as around 330–490, 600–640, 750–950 and 1140–1400 nt, which could be a common feature^10^. These fragments in vRNA5 form local motifs or interact with other RNA region (Fig. 2).

Low NP binding regions are accessible for inter- and intramolecular RNA interaction vital for the virus replication cycle. Indeed, it was shown in different studies that synonymous mutations that abolish predicted RNA secondary structure motifs result in attenuation of IAV replication^18,21,22,45^. Identified vRNA5 structural motifs, especially in low NP binding regions, could be crucial for packaging and/or replication.

The determined secondary structure of vRNA5 coupled with isoenergetic microarray mapping led to designing of effective antisense oligonucleotides. Few papers report antisense oligonucleotides that target vRNA5 and inhibit virus replication^46–48^. Moreover, only the *panhandle* region and nearby 3′ end regions were targeted so far. Therefore, for the first time, interior regions of vRNA5 were effectively targeted. Also, modified RNA oligonucleotides (2′OMeRNA and 2′OMeRNA-LNA) were used here, confirming their usefulness in antisense strategies using a low dosage of oligonucleotide.

Most ASOs influenced virus replication (measured by IFA; Fig. 7), which show the final effects of inhibition on the levels of virus particles. From fourteen antisense oligonucleotides, five significantly inhibited virus replication from 43 to 88%. Simultaneously, the amount of viral RNA is decreased, but to a lesser extent: up to 55% for 883–11L (Fig. 8). Only for 538–10L, which does not inhibit influenza, this trend reversed: 14% reductions in vRNA correspond to higher NP levels via IFA. Both observations show that the inhibition process is complex and probably involves deregulation of vRNA5 functions. Different inhibition effect of designed ASOs is reasonable and indicated various importance of target regions and maybe also vRNA5 different accessibility resulting from interactions with, for example, other vRNA or viral and cellular proteins. Importantly, the control oligonucleotides, NEG, MX did not influence influenza replication, including 1079–12M which targeting double stranded region (Figs 7 and 8).

It is notable that the three best inhibitory oligonucleotides (883–11L, 474–21M, 1253–13M) have target region fragments found to have low *in vivo* NP binding^10^. These confirm the effectiveness of the applied approach where detailed structural *in vitro* studies of vRNA lead to the detection of important for viral cycle motifs whose functions could be blocked by antisense oligonucleotide binding.

The most effective antisense oligonucleotide is 883–11L, which targets region 878–888 nt (Figs 6–8). Interestingly, this is in the larger fragment around 760–920 nt that was shown to be relatively free of NP binding *in vivo*^10^. Oligonucleotides 883–11L and 883–11M may affect a possible interaction between vRNA5 and vRNA2. Nucleotides 874–883 in vRNA5 are complementary to region 25–34 nt in vRNA2: the binding of 883–11M and 883–11L to nts 878–888 nt would preclude this putative vRNA-vRNA interaction. The highest inhibition, observed for 883–11L vs. 883–11M could come from more stable duplex with target RNA for LNA modified oligonucleotide.

Also, in the low abundance *in vivo* NP region, is located the target site for 474–21M and 474–21L oligonucleotides (465–485 nt)^10^. Surprisingly, higher inhibition was observed with 2′OMeRNA 474–21M (64%) than the same oligonucleotide containing LNA modifications 474–21L (31%) (Figs 6, 7). Inhibition suggests that the conserved hairpin 460–476 nt (conservation 83.3%) is important for influenza virus replication. (Fig. 5, Supplementary Data 3). Oligonucleotides 474–21M and 474–21L may also interact with region 750–755 nt and this could influence inhibition results (Table S5).

Among low NP binding fragment 1140–1400 nt^10^ antisense oligonucleotides 1253–13M, 1253–13L and 1333–16L (Fig. 6) have binding sites and their inhibitions were statistically significant (Figs 7, 8). 1253-13M, 1253–13L inhibited IAV replication similarly, by 48% and 43%, respectively. The ASOs binding region is located within low structured region therefore, this could be a reason that incorporation of LNA modifications did not improve its inhibitory properties. 2′-O- methyl RNA can form duplex that is thermodynamically stable enough to unwind the weak helix 1255–1257 nt/1264–1266 nt.

Antisense strategies targeting vRNA5 secondary structure conserved motifs could be powerful alternative therapies against influenza; especially for fast spreading pandemic and deadly strains. Important vRNA5 regions and structural motifs revealed by antisense oligonucleotides could be considered alongside other anti-influenza inhibitors (e.g. small molecules) for combined treatments. Also, vRNA5 fragments accessible to oligonucleotides could be used for techniques applying complementary oligonucleotides: e.g. *in cell* labeling, detection or viral RNA isolation through hybridization. Moreover, secondary structure of vRNA5 might be utilized for the design mutant viruses for research and/or attenuated influenza vaccines.

## Methods

### Oligonucleotides synthesis

The synthesis of oligonucleotides was performed via MerMade 12 solid phase synthesis with phosphoramidites. All oligonucleotides (DNA, modified DNA, 2'OMeRNA, 2'OMeRNA-LNA) were deprotected according to published procedure^49,50^. To solid support was added 32% ammonia solution and incubated in 55 °C, 18 h, than evaporated and dissolved in water. Primers for reverse transcription, contained C6-aminolinker at 5′-end, were treated, in next step, with 80% acetic acid (3 h), precipitated in 1% sodium perchlorate solution in acetone and labelled with 5-FAM, 6-JOE, 5-ROX or 6-TAMRA (Anaspec). 300 µg of oligonucleotide was dissolved in water (11 µl) and 75 µl of 0.1M sodium tetraborate, pH 8.5 and fluorophore [TAMRA, FAM (200 µg) or JOE, ROX (250 µg)] in 14 µl DMSO was added. Reactions were incubated at 23 °C for 18 h by slowly mixing (150 rpm) then precipitated and run on 12% denaturing PAA gel. Oligonucleotides for ribonuclease H assay were purified using thin layer chromatography (TLC) and mobile phase was n-propanol/ammonia/water (52/35/13). Antisense oligonucleotides (2′-O-methyl RNA and 2′-O-methyl RNA containing LNA modifications (LNA - *locked nucleic acids*) were purified with TLC (short oligonucleotides) or denaturing 12% PAA gel (oligonucleotides longer than 11 nt). Oligonucleotides concentration was measured at 260 nm with Nanodrop spectrophotometer (Thermo Scientific). Molecular weights were confirmed by mass spectrometry (MALDI TOF, Autoflex, Brucker).

### RNA preparation

DNA template of A/Vietnam 1203/2004 (H5N1) segment 5 vRNA (vRNA5) was obtained from the pHH21 plasmid. For PCR were used PR1 and forward PR2 (with T7 promoter sequence) (Table S1). Transcription was performed using AmpliScribe^TM^ T7-*Flash*^TM^ Kit and then RNA was purified with RNeasy MinElute Cleanup Kit. The yield was measured with Nanodrop**®** spectrophotometer at 260 nm and quality of RNA was examined by agarose gel (1%) electrophoresis at 4 °C and with comparison with RiboRuler High Range RNA Ladder.

Prior to chemical probing or hybridization to isoenergetic microarrays, the vRNA5 solution was folded by heating in buffer 1 (300 mM NaCl, 5 mM MgCl_2,_ 50 mM HEPES pH 7.5) or buffer 2 (300 mM KCl, 5 mM MgCl_2_, 50 mM HEPES pH 7.5) at 65 °C for 5 min and cooled down to room temperature. Appropriate folding to one native structure was checked on 1% agarose gel (Fig. S1).

### Chemical mapping

Before chemical mapping 2 µM RNA was folded in buffer 1 as described above. Next, vRNA5 was treated, depending on regent, with 3.3 mM NMIA for 40 min., 9.5 mM CMCT for 30 min., 1.6 mM DMS for 15 min. or 1.5 mM kethoxal for 20 min. In case of kethoxal mapping after reaction 1 μl of 0.35M potassium borate was added. Controls were performed in the same conditions but without mapping reagent. The reactions were performed at 23 °C or 37 °C. Reactions were stopped by ethanol precipitation.

### Chemical mapping read-out by reverse transcription

Chemically modified nucleotides were identified by reverse transcription. Primer extension was performed with six primers separately (P1, 2L, 3L, P4, P5, P6 –Table S2), with SuperScript III Kit (Invitrogen). Primers for reverse transcription were labelled with FAM, JOE (control and reaction), ROX and TAMRA (ddNTP sequencing ladders). Reverse transcription reactions were performed according to Invitrogen protocol. Briefly, probes of RNA, FS buffer and primer (final concentration of 2 µM) were heated for 3 min at 90 °C, then annealing was performed for 10 min at 55 °C and probes were placed on ice for 4 min. Then a mix of dNTP, DTT, buffer and enzymes was added to get final concentrations: 2 μM primer, 1x FS buffer, 1.2 mM each dNTP, 6 mM DTT, 50U SuperScript III, 4U RNasin®. Probes (including controls) were incubated at 55 °C and after 50 min precipitated in ethanol with 0.3M sodium acetate. All probes were next suspended in HiDi Formamide (Applied Biosystems) and mixed with appropriate ddNTP sequencing ladders (mostly performed with 2′-3′-dideoxy-TTP for ROX and 2′-3′-dideoxy-CTP for TAMRA labelled primer) and separated by capillary electrophoresis (Hitachi Applied Biosystems 3100 Avant).

### Chemical mapping data analysis

ABI files were analyzed with ShapeFinder software^51^. Quantitative reactivities for individual chemical mapping datasets were normalized to a scale in which 0 indicates an unreactive site and the average intensity at highly reactive sites is set to 1.0. For normalization first 2% of the highest reactivity values were excluded from individual dataset and the average for remaining 8% highest reactivities were calculated. The reactivity values at all nt were normalized dividing by this average. Reactivities ≥0.700 are considered as strong, 0.500–0.700 as medium and <0.500 as weak. Nucleotides with no data were indicated as −999. Normalized reactivities from each primer extension reaction were processed independently. For each mapping (SHAPE, DMS, CMCT, kethoxal) at least three datasets were obtained from each primer and the average of results was used for prediction of secondary structure.

### Secondary structure prediction

The secondary structure of vRNA5 was generated using RNAstructure 5.7 software^52–54^ with incorporated experimental data^55^. From CMCT, DMS and kethoxal mapping data only reactivities ≥0.700 were used as constraints (mode: “Chemical modification”). At the same time NMIA reactivities from SHAPE mapping as pseudoenergies were implemented in prediction (mode: “Read SHAPE Reactivity - Pseudo Energy Constraints”, using text file with calculated reactivities for each nucleotide). The Slope and Intercept parameters were 1.8 and −0.6, respectively^56^.

### Bioinformatic analysis of base pairs probabilities

The vRNA5 base pair probabilities were obtained using RNAstructure^52^ program implemented in RNAstructure Web Server for RNA structure Prediction (Predict a Secondary Structure mode). The chemical mapping experiments results were incorporated and all parameters were as described in *Secondary structure prediction* section.

### Bioinformatic analysis of structure conservation

All available vRNA5 sequences were obtained from the NCBI Influenza Virus Resource^57^. The coding RNAs were reverse transcribed in silico then aligned using MAFFT (FFT-NS-1 method)^58^. vRNA5 structure models were mapped to the alignments then preservation of base pairing was calculated to give the percent conservation of canonical base pairing (GC, AU and GU pairing) as well as a measure of inconsistent, or potentially non-canonical, pairs are model base pair sites. Swaps between canonical pairing types were manually inspected to identify potential structure-preserving (consistent or compensatory) changes.

### Isoenergetic microarrays preparation

Isoenergetic microarrays were similarly as described previously^59,60^. Microarray slides (Silane-Prep Slides, Sigma-Aldrich) were coated with 2% agarose, reduced with NaIO_4,_ washed in water and dried overnight. Probes for isoenergetic microarrays are 2′-O-methyl-LNA penta- and hexamers containing selected 2,6-diaminopurine riboside modification (LNA or 2′-O-methylated nucleotide)^30,31,61^. All probes comprising C6-aminolinker at 5′ and were printed in three repeats with Nanoprint^TM^ at 50% humidity. The final concentration of probes was 0.1 mM and printing buffer was 3xSSC, 0.05% SDS, 0.001% CHAPS. List of complementary probes to vRNA5 is in Supplementary Data 2. The negative controls were UUUUU (2′OMeRNA) and printing buffer. After printing, microarrays were incubated at 37 °C in 100% humidity chamber for 12 h and next treated with NaBH_4_ (35 mM) solution in ethanol and 1x PBS buffer (1:3 v/v). Then, isoenergetic microarrays were washed of reducing reagent in water (3 times) and dry.

### Hybridization to isoenergetic microarrays

Microarray mapping using isoenergetic microarrays was conducted similarly as described before^20,62^. For experiment vRNA5 was labelled with [α^32^P] ATP during *in vitro* transcription and purified with RNeasy MinElute Cleanup Kit. Upon every hybridization experiment vRNA5 was diluted in buffer 1 or buffer 2 and final concentrations of vRNA was 2 μM.

Radioactively labelled vRNA5 was folded in selected buffer (buffer 1 or buffer 2) as described above. Hybridization was performed with 300 000 cpm vRNA5 on microarray slide at 100% humidity chamber. The final volume was 250 μl and incubation was performed for 18 h at both 37 °C and 23 °C. Then microarrays were washed (1 min and temperature of buffer was 37 °C), dried and after exposure to imaging screen scanned by Fujii phosphoimager.

Quantitaive analysis were performed with ArrayGauge V2.1. Binding intensities were denoted as strong, medium and weak when the integrated spot intensity was ≥1/3, ≥1/9 and ≥1/27 of the strongest integrated spot intensity, respectively. The binding site was named from the number of RNA middle nucleotide, complementary to the probe sequence.

### RNAse H assay

vRNA5 (3 μM) was folded and next incubated with appropriate short DNA oligonucleotide (3 μM) in buffer 1 with 1 mM DTT, then 5U of ribonuclease H was added. The reaction was carried out at 37 °C for 30 min. The reaction was stopped by heating for 10 min at 65 °C. Then, precipitation and primer extension were performed as described above.

### Cells experiments with oligonucleotides targeting vRNA5

MDCK were obtained from Sigma-Aldrich. Growth medium was DMEM with 10% fetal bovine serum (FBS), 1x PSG (2 mM L-glutamine, 100 U/ml of penicillin and 100 µg/ml of streptomycin) and cells were cultured at 37 °C in a 5% CO_2_ humidified incubator. Influenza virus A/California/04/2009 (H1N1) was a gift from Prof. Luis Martinez-Sobrido, University of Rochester. Virus titers were determined with standard plaque assays^63,64^.

24 h before experiment with antisense oligonucleotides 2.5 × 10^6^ cells were seeded in 10 cm^2^ plate. Prior to transfection 0.5 μM of each oligonucleotide was vacuum-dried, than dissolved in OPTIMEM**®** medium (Life Technologies). Transfection was carried out with Lipofectamine 2000 according to manufacture instruction. The experiment was performed with both 96-well and 24-well plates. In the case of 96-well plate experiment the final Lipofectamine 2000 concentration was 0.4 μl/well. During incubation of oligonucleotide with Lipofectamine 2000 cells were passaged and after 25 min they were added to OPTIMEM®-Lipofectamine-oligonucleotide mix. Cells with oligonucleotide were seeded on 96-well plate (2 × 10^4^ cells/well) and after 12 h the medium was changed for the standard growth medium and incubated at 37 °C in a 5% CO_2_ humidified incubator for the following 6 h. For 24-well plate experiment all reagents were scaled up proportionally to 96-well plate experiment.

Next, cells were infected with influenza virus. In details, 18 h after transfection MDCK cells were washed with 1x PBS (sodium chloride 137 mM, phosphate buffer 10 mM, potassium chloride 2.7 mM) pH 7.4 and infection was conducted in infection medium (1x PBS with 0.3% bovine serum albumin (BSA)). MDCK were infected with wild-type virus A/California/04/2009 (H1N1) at MOI of 0.001. After 1 h of infection at room temperature medium of 0.3% BSA, 1x PSG, TPCK-trypsin 1 μg/ml in DMEM was added. Plates were incubated at 33 °C in a 5% CO_2_ in a humidified incubator. Supernatants with virus were collected after 24 h. Virus titer of A/California/04/2009 (H1N1) was calculated with the Indirect Immunofluorescence Assay (IFA). Additionally, for selected oligonucleotides, qRT-PCR was used for calculation of vRNA level.

### Indirect immunofluorescence assay (IFA)

96-well plates were infected with thawed supernatants in 10-fold dilutions. 8 h after infection the fix and permeabilization solution was added (0.5% Triton X-100, 4% formaldehyde, 1x PBS pH 7.4). Incubation was carried out for 15 min at room temperature. Then cells were washed with 1x PBS and blocking buffer was added (3% BSA in 1x PBS pH 7.4) and plates were incubated for 12 h at 4 °C. Blocking buffer was aspirated and solution of antibody (Anti Influenza A Antibody Nucleoprotein Clone a1, Merck Millipore) 100x diluted in the blocking buffer was added (45 μl/well). After 2 h of incubation at 33 °C, the solution was aspirated, plates were washed 3x with 1x PBS and rabbit anti-mouse IgG antibody (1 mg/ml in the blocking buffer) with FITC (Merck Millipore) was added. Plates were incubated for 2 h, washed with 1x PBS, visualized under the fluorescence microscope and FFU/ml was calculated.

### qRT-PCR

For determination of viral RNA amount the qRT-PCR was used according to described methods with changes^36,65^. Total RNA was isolated from infected cells. 24 h after infection (performed on 24-well plate) 500 μl of TRIZOL reagent was added on every well. RNA was isolated with Chomczynski Sacchi method protocol^66^. 500 ng of prepared probes were treated with 2U of DNase I for 30 min at 37 °C and RNA quality was estimated with agarose gel electrophoresis. Next, 2 μl of prepared RNA was used for reverse transcription with 1pmol of primer RT (Table S2). The reaction was performed with SuperScriptIII (Invitrogen) at 55 °C for 50 min. From 10 μl of reaction 1 μl of cDNA was used as a template for qRT-PCR. The reaction was carried with primers QR and QF (Table S3). Oligonucleotide Q was TaqMan probe. The reaction was performed according to BioRad protocol. The standard for qRT-PCR was vRNA7.

### Test of ASO cytotoxicity

To evaluate cytotoxicity of oligonucleotides MTT [3-(4,5-dimethylthiazole-2-yl)-2,5-diphenyltetrazolium bromide] assay was carried out. MDCK cells were seeded in 96-well plates (20,000 cells per well) and were grown up to 80–90% confluency. Than transfection was conducted with 0.5 μM of oligonucleotide. The control was Lipofectamine 2000 solution. After 12 h MTT (5 mg/ml) in DMEM was added and plates were incubated for 2 h. The medium was removed and DMSO (100 µl per well) was added. Plates were put on the rocker (slow movement) for 15 min and absorbance was measured at 570 nm (xMark Microplate Spectrophotometer, BioRad). Cell viability was presented as the percentage ratio of absorbance of oligonucleotide well to the absorbance of control well.

## Supplementary information

Supplementary Information

Supplementary Data 1

Supplementary Data 2

Supplementary Data 3
