# Supplementary material for: Secondary structure of the segment 5 genomic RNA of influenza A virus and its application for designing antisense oligonucleotides
Source: Sci Rep. 2019 Mar 7;9:3801. doi: 10.1038/s41598-019-40443-7 (PMC6406010; doi:10.1038/s41598-019-40443-7)
Supplement: Supplementary file 1 — Supplementary Information [file 41598_2019_40443_MOESM1_ESM.pdf]

SUPPLEMENTARY INFORMATION

Secondary structure of the segment 5 genomic RNA of influenza A virus and its application for designing antisense oligonucleotides

Paula Michalak[1], Marta Soszynska-Jozwiak[1], Ewa Biala[1], Walter N. Moss[2], Julita Kesy[1], Barbara Szutkowska[1], Elzbieta Lenartowicz[1], Ryszard Kierzek[1], Elzbieta Kierzek[1]*

[1]Institute of Bioorganic Chemistry Polish Academy of Sciences, 61-704 Poznan, Noskowskiego 12/14, Poland

[2]Roy J. Carver Department of Biophysics, Biochemistry and Molecular Biology, Iowa State University, Ames, IA 50011, United States of America

*Corresponding author: E-mail: elzbieta.kierzek@ibch.poznan.pl, Institute of Bioorganic Chemistry Polish Academy of Sciences, 61-704 Poznan, Noskowskiego 12/14, Poland, Tel.: +4861-853-8503; Fax: +4861-852-0532

Name	Sequence 5’→3’
PR1	GCGTAATACGACTCACTATAGGGAGTAGAAACAAGGGTA TTTTTC
PR2	AGCAAAAGCAGGGTAGATAATC

Table S1. Primers for PCR for obtaining vRNA5 DNA template.

Name	Complementary vRNA5 region (nt)	Sequence 5’→3’
P1	1541-1565	AGCAAAAGCAGGGTAGATAATCAC
2L	1306-1331	GAgAGaATGgTACtCTCtGCAtTTG
3L	1043-1063	CTcTcGTgCGtACtGGaATG
P4	784-803	GATCAAGTGCGAGAGAGCAG
P5	522-544	GTCAATTAGTGTGGATGGCATG
P6	263-282	GGTCCAGAGAAACCTTCCC
P7	1269-1287	CCTGGAAGAACACCCCAG
P8	1092-1116	CCTGATGATATGGCATTCCAATCTA

Table S2. DNA primers for reverse transcription. LNA is marked with small letter. All primers were 5'-labelled with JOE, FAM, TAMRA or ROX.

Name	Sequence 5’→3’	Length (nt)
QF	AGACCAATCTTGTCACCTCTGAC	23
QR	AGGGCATTTTGGACAAAGCGTCTACG	26
QP	TCACCGTGCCCAGTGAGCGAGGACTGCA	27
RT	ATGAGTCTTCTAACCGAGGTCG	22

Table S3. DNA primers for RT-qPCR. Primer QP was labelled with 6-TAMRA at 3’and 5-FAM at 5’ end.

Binding site [a]	Probe sequence [b]	Nucleotide of RNA target complementary to 3'g of hexamer probe [c]	ΔG°$_{37}$ (kcal/mol) for duplex modified probe/vRNA5 [d]		Hybridization result [e, f] buffer 1, 37°C	Hybridization result [e, g] buffer 2, 37°C
20/114/891	GdDdDg	U, C, A	-7.79	-9.81*	m	
21/273/783	dGdDdg	U, G, A	-9.00		w	m
22/184/355/ 880/883/1251/ 1280/1300/ 1429	dDgDdg	U, A, G, G, C, C, G, C, A	-9.24	-11.37*	s	m
23/257/815/ 1182/1301	dDdGag	U, C, C, C, U	-8.39	-10.19*	m	s
30/48/472/958 /1185	GdCdDg	A, A, C, A, U	-8.62	-10.64*	m	
33/654/864/ 1365/1491	UdUgDg	G, G, C, U, U	-8.47		m	w
36	GdGUdg	A	-9.20		m	
37/135	GgDgUg	U, G	-10.82			m
38/999/1170/ 1178/1224	DgGdG	A, U, C, U, U	-9.46		m	w
41/276/324/ 439/478/1074	CdGdGg	C, U, C, C, C, U	-9.86	-12.44*	m	m
42/215/325/ 371/785	GCdGdg	C, U, C, A, U	-10.44	-12.57*	m	w
50/1220	GdGdCg	U, C	-9.84	-11.92*	m	w
51/899/998/ 1143/1177/ 1223	GgDgAg	G, C, A, C, A, C	-10.72	-12.47*	m	m
52/343/1227	CgGdG	U, C, C	-9.52		m	m
53/120/937/ 1228	UCgGdg	C, G, A, C	-10.23	-12.36*	w	m
54/938	uUcGgg	U, U	-9.28		m	
66/717/802/ 920	GGdUcg	A, U, U, U	-9.63			m
68/874/1036/ 1260/1297	dDgGdg	A, A, A, G, U	-10.59		m	m
69/639/1298/ 1362	GdAgGg	U, C, U, C	-9.41	-11.99*	m	m
70/760/1363	uGdDgg	C, U, C	-9.17	-12.1*		m
71/78/1304/ 1364/1490	dUgDdg	C, A, U, C, G	-9.11	-11.24*	m	m
105/116	DcGdDg	G, U	-9.22	-11.24*		m
106/915/926/ 1069/1499	dDcGag	U, G, C, C, A	-9.03	-10.83*	m	m
112/840/970/ 1261	dDdGgg	G, C, C, U	-9.43	-12.36*	m	
113/1560	dDdDgg	C, G	-8.08	-11.01*	w	m
117	GDcGDg	U	-8.80	-10.82	m	
136/1359/1410	GGgDg	A, C, C	-9.91			m
157/1394/1484	dGdUgg	A, A, C	-9.66	-12.59*	m	s
158/758	DdGdUg	C, G	-8.53	-10.47*	m	
159/759/881/ 884/1249/1252 /1281/1430	GdDgDg	A, A, U, U, G, U, U, U	-9.74		w	m
160/348/375/ 640/882/1250/ 1299	DgDdGg	U, G, G, C, U, U, C	-9.61	-12.19*	m	m

161/214/349/ 784/1347/1385 /1461	CdGdDg	C, G, C, U, G, G, A,	-9.07	-11.09*	m	w
171/796/1269	DgUgCg	G, C, C	-10.74	-12.82*	m	
172/797	dDgUgg	G, G	-9.44		w	m
174/258/1262/ 1302	GdDdGg	A, U, C, U	-8.58	-11.16*	m	m
175/228/339/ 836/1214/1263 /1480	gGdDdg	C, A, G, A, A, C, G	-9.56	-11.69*	w	m
176/622/886/ 1048/1283/143 2/1481	uGgDdg	U, A, C, A, C, C, U	-9.97	-12.10*	w	m
177/399/408/ 804/887/948/ 1044/1145/ 1482	dUgGdg	U, G, C, A, U, C, G, C, U	-10.46	-12.59*	m	s
178/529/805/ 949/1483	GdUgGg	U, G, U, U, U	-10.04		w	m
179/806/950/ 1022/1087/ 1112/1306	uGdUgg	C, C, C, G, G, U, U	-9.04	-11.97*	w	m
185/356/547	uDdGdg	U, U, C	-8.78	-10.91*	m	w
201/416/563/ 863	dUgDgg	C, U, U, U	-9.9	-12.83*	w	m
216/241	GGCdGg	U, G	-9.64		m	w
218/321/1443	AgGgC	U, U, U	-10.01			m
200/220/505	UGdGgg	U, C, U	-9.99	-12.92*	w	m
221/378/723/ 1451	CuGdGg	C, U, U, U	-9.62	-12.20*	m	
229/340/1296	DgGdDg	U, U, G	-10.17			m
230/311/1171	CdGgAg	U, G, C	-10.05	-11.80*	m	m
244/1106	UdUgGg	G, G	-8.90		m	
256/457/469/ 546/814/1181	dDgDgg	G, C, A, A, G, C	-10.03	-12.96*	s	m
265	UcCcUg	A	-10.47			s
266	UUcCcg	A	-9.17			s
272/338/1479	GdDdCg	G, U, A	-8.13		m	
274/376/414/ 561/641/859/ 1072/1327	GdGdDg	U, C, G, A, C, A, G, A	-9.50	-11.52*	m	m
275/642/644/ 790/860/1073/ 1203/1221/ 1328/1330	dGdGdg	U, U, C, C, U, U, C, G, U, C	-10.71	-12.84*	s	m
291/1118	CCcDC	A, G	-7.72			m
307/1292/1386 /1396/1486	dCdGdg	A, A, U, A, A	-9.83		m	w
308/452	GdCdGg	U, G	-9.41		s	m
377/415/562/ 646/722/862/ 1205/1450	uGdGdg	U, U, U, C, C, C, C, A	-10.09	-12.22*	s	s
395	DcUcCg	U	-10.04		w	s
400/888/955/ 1045/1146/ 1211/1324/ 1426	DdUgGg	U, U, A, U, U, C, A, A	-9.12	-11.70*	w	m
411/849/1524	dDcDug	C, G, G	-7.22		m	
418/565/889/	dDdUgg	U, U, C, U, C	-7.95	-10.88*		m

1090/1212						
458/470	cDdGdg	C, C	-9.49	-11.62*	m	m
461	uCcCa	U	-8.86		w	s
468/495	DgDgUg	C, G	-10.26	-12.20*	m	m
471/876	DcDdGg	U, C	-8.73	-11.31*	m	
492/578/1513	gUcUcG	U, U, U,	-9.63		m	
496/1331	UdGdGg	A, U	-9.15			m
530/935/1034	GGdUgg	C, U	-9.69	-12.62*	w	m
532/659	GUgGdg	A, A	-10.39		m	m
533	uGuGgg	U	-9.87		w	m
534/683	gUgUgg	C, A	-9.77	-12.70*	s	
535	DgUgUg	C		-11.61*	s	
623/1433	uUgGdg	U, U	-9.41			m
435/966/639/ 965/1553	GgGuAg	A, U, C, C, C	-9.79	-11.54*	w	m
638/839/969/ 1016/1361	dDgGg	A, U, C, A, U	-9.52		m	w
643/645/721/ 789/791/861/ 898/1066/1142 /1202/1204/ 1222/1329	GdGdGg	U, U, C, G, U, U, C, G, C, G, U, U, U	-10.29	-12.87*	m	
657	gGdUdg	A	-9.83		w	m
669	GCdGug	C		-11.92*	m	
677	DcGgDg	G	-10.81		s	s
679/1189	GUdCgg	C, U	-8.68	-11.61*	m	
614/726/779/ 846	dUcCug	A, C, C, G	-8.74	-11.82*		m
775/1411	UgGgAg	U, C	-10.53	-12.28*	w	m
787/812	GdGCdg	C, U	-10.44	-12.57*	m	w
792/1067	CGdGdg	C, C		-12.37*	w	m
816/841/1183	CdDdGg	U, C, U	-8.15	-10.73*	m	m
875/1017/1037 /1509	CDdGgg	U, C, U, G	-9.39	-12.32*	m	m
885/1282/1431	GgDdGg	U, U, U	-10.17		w	m
901/992/1049/ 1244/1284	CUgGdg	C, C, U, C, U	-10.37	-12.5*		m
909	CgGdAg	A	-9.74		s	
924/1001	CgDgG		-9.76		m	
925/1068	DcGdGg	C, U	-10.01	-12.59*	m	m
936	CgGdUg	C		-12.12*	m	w
952/1207	GgUgDg	A, C	-10.74	-12.76*	m	w
475/838/922/ 961/1360	AgGgAg	G, U, A, G, C	-10.34	-12.09*		s
963	gUdGgg	C		-13.09*	w	m
982	CgGuGg	G	-10.37			m
1013	GgUcDg	U	-10.25		m	
1018	GcDdGg	C		-12.45*	s	
1077	UdUcDg	C		-10.00		m
1172/1445	UCdGgg	U, C	-9.50	-12.43*	w	m
774/837/1215/ 1264/1469	GgGdAg	A, U, U, U, G	-10.24		w	m
1241/1408	GDgGUg	G, A	-9.24		m	
1253	CGdDgg	U	-9.32			m
795/1056/1270	gUgCg	U, A, G	-9.48		m	
1272	CCcDG	A	-8.17			m
1273	CcCcA	C		-9.88*		m
1322	UgGUdg	G	-9.25		m	

1371/1533	dGuGdg	G, G	-10.06		m	
1374	CUcDgg	A	-9.32		m	
536/ 1420	UdGuGg	A, C	-8.50	-11.08*	w	m
964/1552	GgUdGg	C, U	-9.98	-12.56*	m	w

Table S4. Isoenergetic microarrays probes that bind strongly and moderately to vRNA5 and their thermodynamic properties. a – binding sites of probes (all complementary possible targets), sites are denoted by the middle nucleotide of the complementary RNA region to specific pentamer; b - nucleotides in capital letter (A, C, G, U, D) are 2'-O-methyl-RNA nucleotides, in small letter (a, c, g, u, d) are LNA nucleotides, D and d are 2,6 –diaminopurine (2'-O-methyl type or LNA, respectively); c - listed nucleotides concern each binding sites in column 1, respectively; d- ΔG°_{37} calculated as modified probe/RNA duplex for listed binding sites [1, 2], * - ΔG°_{37} calculated for duplex of full complementary hexamer probe (3'g of probe is paired with C of RNA target); e - binding was considered strong (s), medium (m) and weak (w), when the integrated intensities were ≥1/3, ≥ 1/9 and ≥ 1/27 of the strongest intensity, respectively; f - hybridization condition: buffer 1 (300 mM NaCl, 5 mM $MgCl_2$, 50 mM HEPES pH 7.5), 37°C; g - hybridization condition: buffer 2 (300 mM KCl, 5 mM $MgCl_2$, 50 mM HEPES pH 7.5), 37°C.

Name	Complementary vRNA5 region (nt)	Sequence 5'→3'	Sites of RNase H cleavage
H1	72-83	CATGAATAAT	71 (*w*)<br>76 (*w*)<br>78 (*w*)<br>82 (*w*)
H2	464-472	AAGAGTGG	681-683 (*s*)<br>471-472 (*w*)
H3	466-485	TTCATAAGAGGAAAGAAAGT	750 (*w*)<br>751-754 (*s*)<br>755 (*w*)<br>470-484 (*s*)
H4	643-651	CTTTGAGAGAG	647 (*s*)<br>649-650 (*s*)
H5	676-684	GTGTACGG	676-684 (*s*)
H6	878-888	TGGACGAAGGA	880-882 (*w*)<br>883 (*s*)
H7	886-895	GCGAAAATGG	890-891 (*s*)<br>893 (*s*)<br>895 (*s*)
H8	1065-1073	AGAACGAGA	1067-1071 (*s*)<br>1073 (*s*)
H9	1102-1109	ATATGGCA	x
H10	1248-1260	GGACCCTAAGAAA	1250-1253 (*w*)<br>1254-1260 (*s*)<br>1261 (*w*)
H11	1257-1264	GAAAGGAC	1261-1262 (*w*)<br>1264 (*w*)
H12	1336-1327	CAATAGAGAG	1331-1332 (*s*)<br>1334 (*s*)
H13	1425-1416	TGGTTAGTGG	1421-1425 (*s*)<br>1426 (*w*)
H14	1415-1427	ATGGTTAGTGGCA	1421-1427 (*s*)<br>1428 (*w*)

Table S5. RNase H assay results for vRNA5 with listed DNA oligonucleotides. *s* - sites of strong RNase H cleavage; *w* - sites of weak RNase H cleavage; x - no cleavage.

Name	Complementary vRNA5 region (nt)	Sequence 5'→3'[a]	ASO type
79-18GP	70-87	cUuUGACATGAGTAAuGa	gapmer
474-21M	465-485	UUCAUAAGAGGAAAGAAAGU	2'OMeRNA
474-21L	465-485	UUcAUAAgAGGaAAgAAAGU	2'OMeRNA-LNA
538-10L	534-543	UCaAUuGGUG	2'OMeRNA-LNA
883-11M	878-888	UGGACGAAGGA	2'OMeRNA-LNA
883-11L	878-888	UGgACgAAgGA	2'OMeRNA-LNA
1069-10L	1065-1074	GAGaACaAGA	2'OMeRNA-LNA
1148-18GP	1140-1157	cAaGCAAACAATGGCgAa	gapmer
1253-13M	1248-1260	GGACCCUAAGAAA	2'OMeRNA
1253-13L	1248-1260	GGACCCUAAGAAA	2'OMeRNA
1333-16L	1326-1341	CAuAACaAuAGaGAGG	2'OMeRNA-LNA
1420-13L	1415-1427	AuGaUUGGUGGaA	2'OMeRNA-LNA
1420-13GP	1415-1427	AuGATTGGTGGaA	gapmer
MX	–	GUUAAGUAAUACAGAGAAGA	2'OMeRNA
NEG	–	AGACCUCUAUAGCAGCU	2'OMeRNA
1079-12M	1074-1085	GCCACAUAUCAG	2'OMeRNA

Table S6. Antisense oligonucleotides (ASOs) designed based on influenza virus vRNA5 secondary structure. NEG and MX are negative controls that are not complementary to vRNA5. MX is scramble oligonucleotide (mixmer) of 474-21M; a – nucleotides in capital letter (A, C, G, U) are 2'-O-methyl-RNA nucleotides, lower case letters (a, c, g, u) are LNA nucleotides, in gapmers the DNA nucleotides are underlined (A, C, G, T).

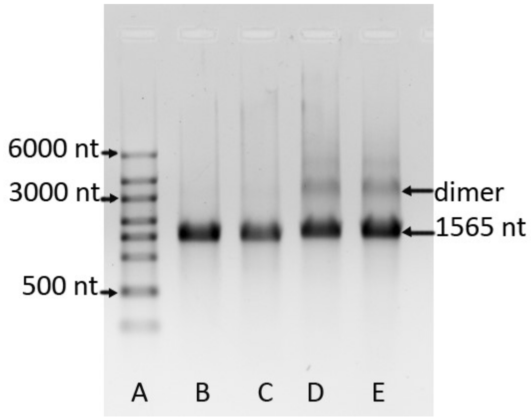

Figure S1. vRNA5 analysis by agarose gel electrophoresis. A – RiboRuler High Range RNA Ladder (Thermo Fisher Scientific), B – vRNA5 after folding in buffer 2; C – vRNA5 after folding in buffer 1; D - vRNA5 in buffer 2 without folding; E – vRNA5 in buffer 1 without folding (see Methods). One structure and no dimer formation was detected for folded RNA in both buffers.

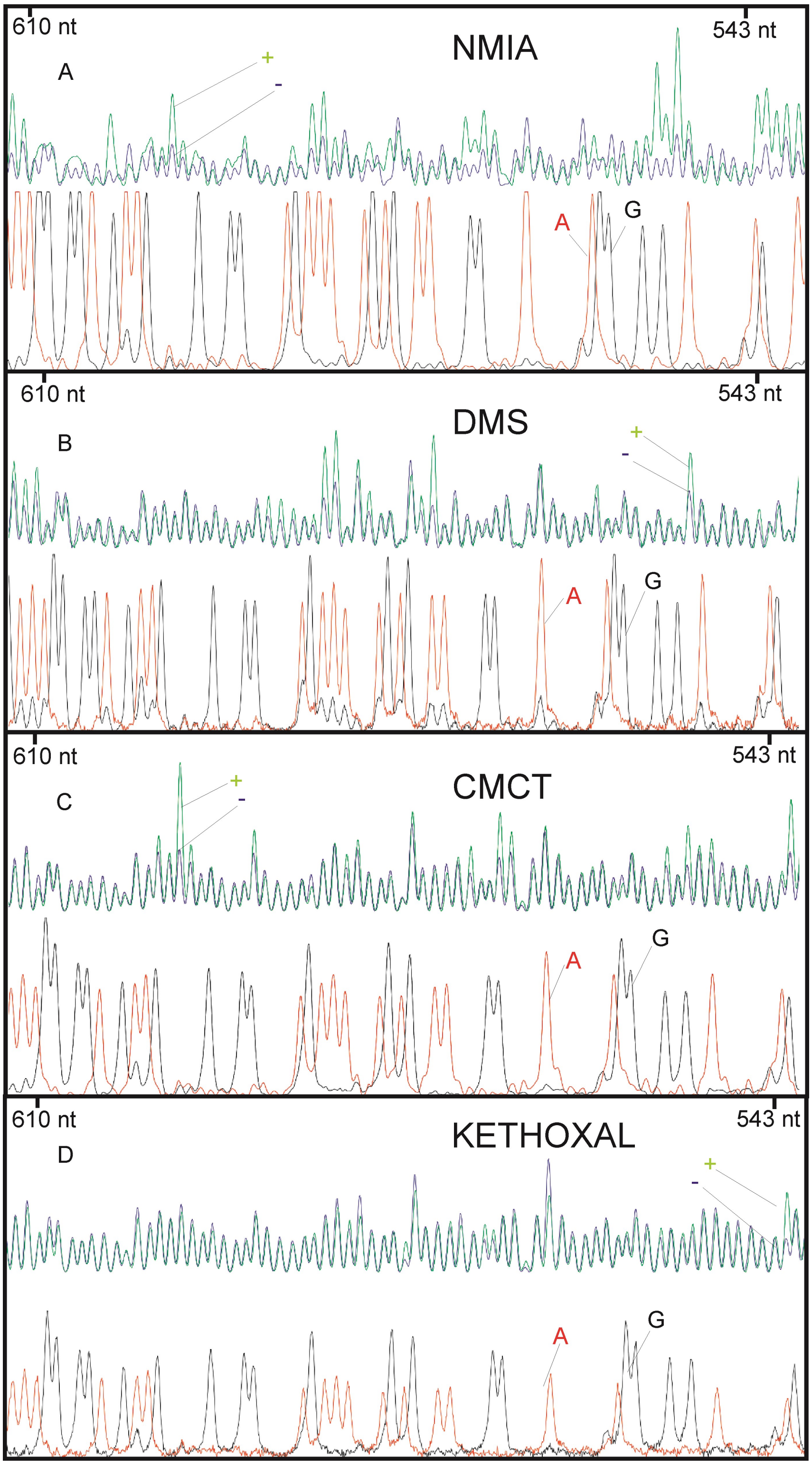

Figure S2. Example of capillary electrophoresis raw data of vRNA5 chemical modification results detected by reverse transcription (using only *Fitted Baseline Adjust* option in ShapeFinder program). On each panel is shown a fragment of SHAPEFinder window with reverse transcription products of chemical mapping with: A – NMIA; B – DMS; C – CMCT; D – kethoxal. The reaction products is a green line (5' labelled with JOE), control is a blue line (5' labelled with FAM). The DNA ladder is respectively a black line - G (5' labelled with TAMRA) and red line -A (5’ labelled with ROX).

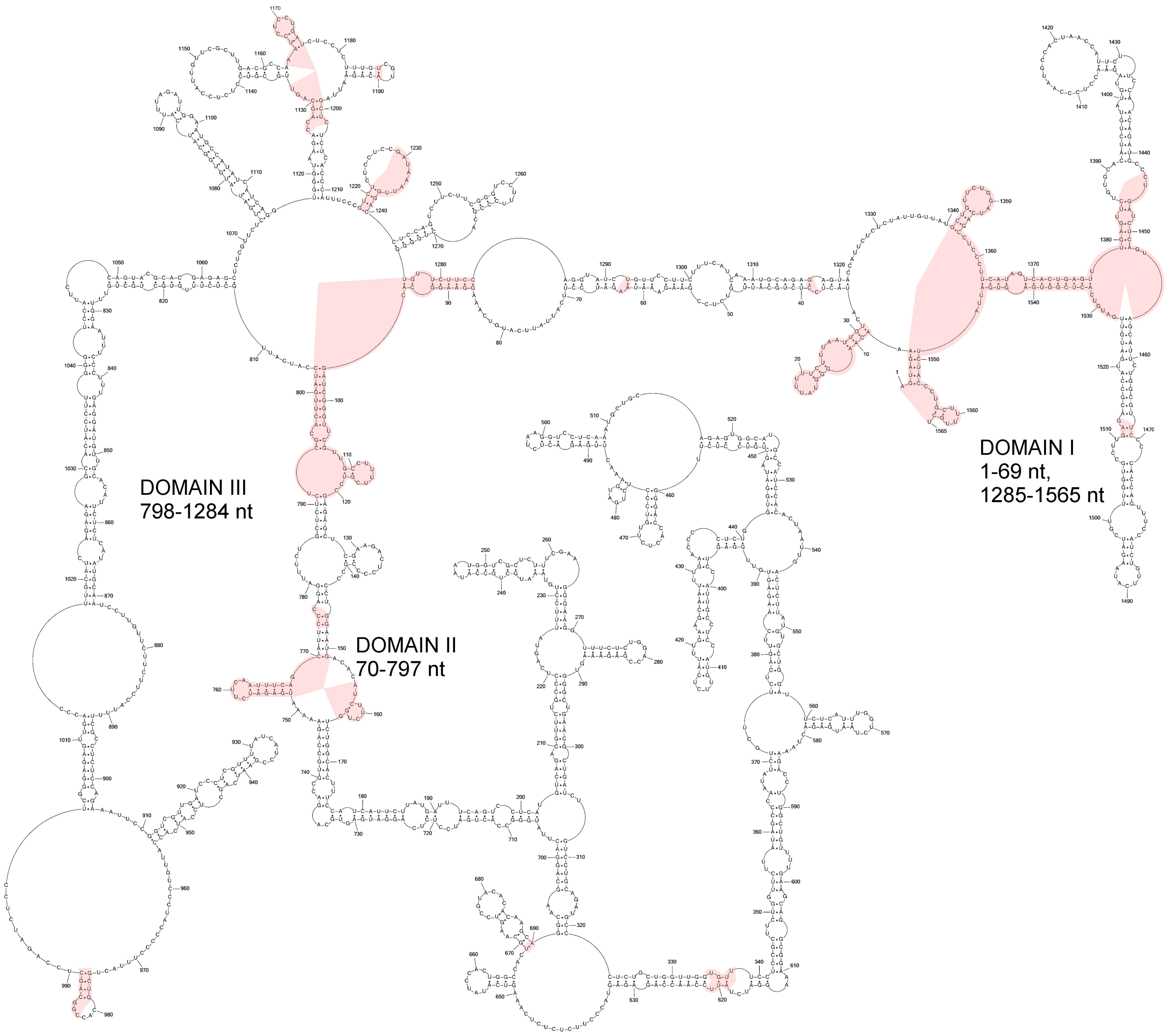

Figure S3. Secondary structure model of vRNA5 predicted with RNAstructure using constrains from chemical mapping at 23°C. Constrains from chemical mapping experiments with DMS, CMCT, kethoxal and NMIA were incorporated analogical as for data from 37°C. The secondary structure motifs that differ from 37°C structure model (Figure 1) are marked with pink colour.

A

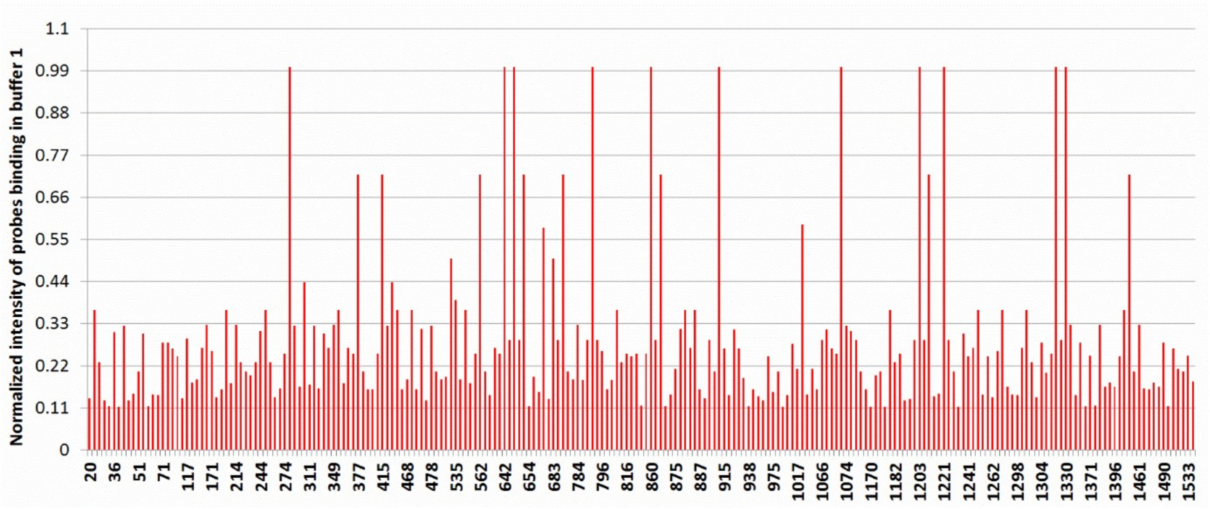

B

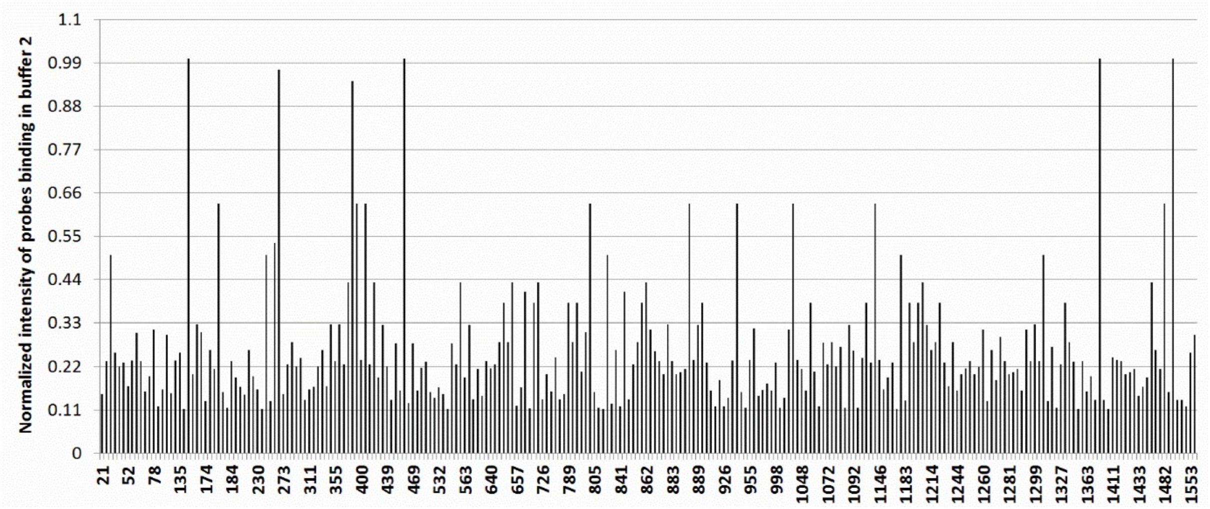

Figure S4. Results of hybridization of vRNA5 to isoenergetic microarrays. All complementary sites for probes that bind strongly or moderately are shown A - in buffer 1 at 37°C; B - in buffer 2 at 37°C. Binding was considered strong and medium when the integrated intensities were ≥1/3 and ≥ 1/9 of the strongest intensity. In graphs the bindings were normalized to the strongest intensity and have values in range 1–0.11, showing bindings: 0.33 ≤ strong and 0.11 ≤ medium <0.33.

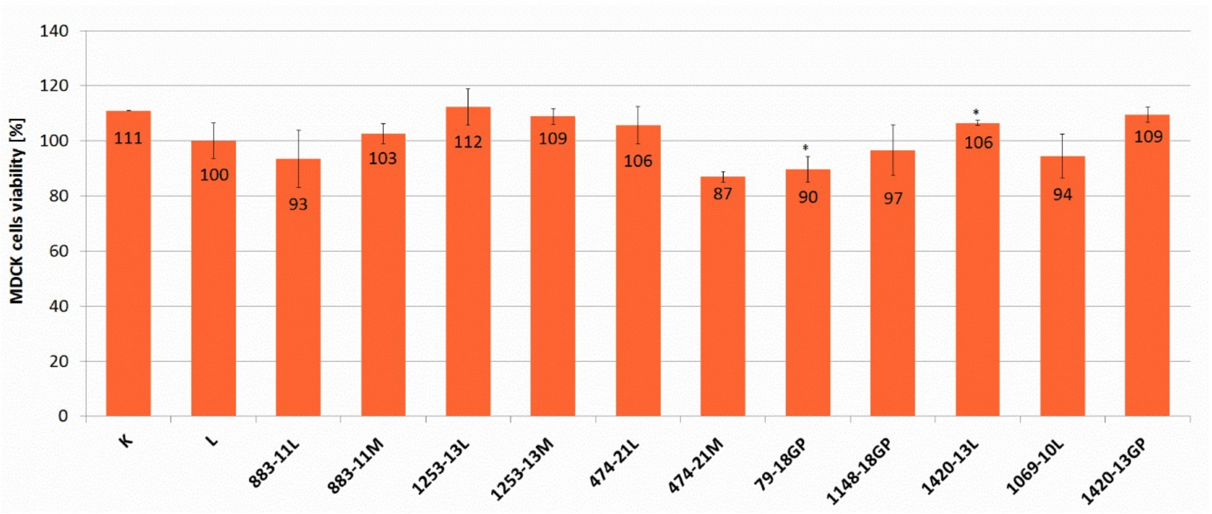

Figure S5. The MTT assay results for MDCK cells treated with 0.5 µM of selected antisense oligonucleotide (ASO). The presented data are average percentages of three biological repeats. K - untreated cells, L- cells treated with Lipofectamine 2000. Statistics were calculated using a two-tailed T-test ($p < 0.01$). Statistically important results are marked with *.

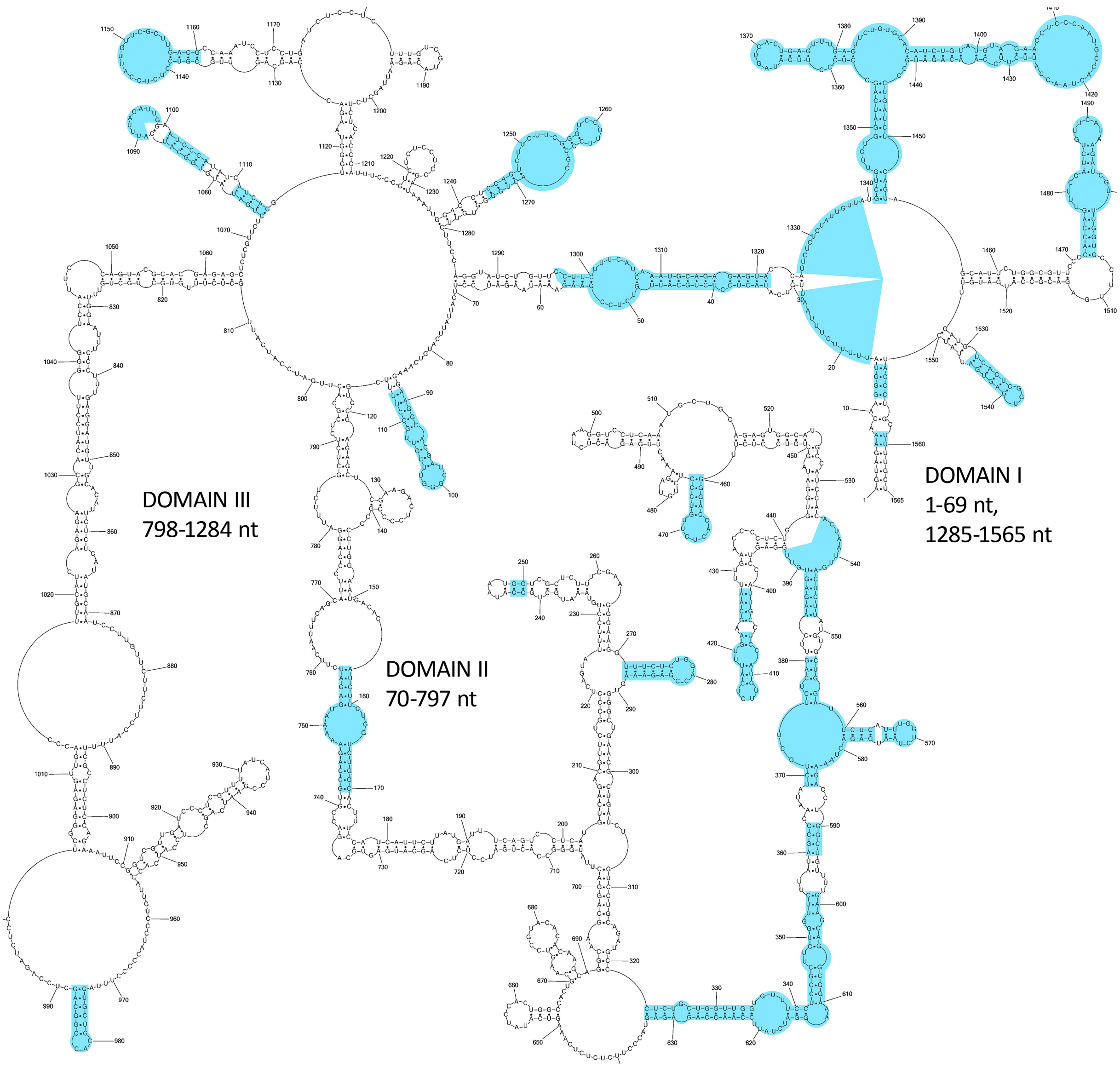

Figure S6. "Mirror motifs" found in determined secondary structures of vRNA5 [(-) strand] and (+)RNA5 [3]. The motifs are marked by blue shadow on vRNA5 secondary structure model predicted by RNAstructure 5.7 using experimental data from 37°C as constraints.

References

1 Kierzek, E., Ciesielska, A., Pasternak, K., Mathews, D. H., Turner, D. H., Kierzek, R. The influence of locked nucleic acid residues on the thermodynamic properties of 2'-O-methyl RNA/RNA heteroduplexes. *Nucleic Acids Res.* **33**, 5082-5093 (2005).

2 Pasternak, A., Kierzek, E., Pasternak, K., Fratczak, A., Turner, D. H., Kierzek, R. The thermodynamics of 3'-terminal pyrene and guanosine for the design of isoenergetic 2'-O-methyl-RNA-LNA chimeric oligonucleotide probes of RNA structure. *Biochemistry* **47**, 1249-1258 (2008).

3 Soszynska-Jozwiak, M., Michalak, P., Moss, W. N., Kierzek, R., Kesy, J., Kierzek, E. Influenza virus segment 5 (+)RNA - secondary structure and new targets for antiviral strategies. *Scientific Reports*, **7**: 15041 (2017).
